# Nutritional Status Indicators as Predictors of Postoperative Complications in the Elderly with Gastrointestinal Cancer

**DOI:** 10.3390/ijerph192013453

**Published:** 2022-10-18

**Authors:** Lucyna Ścisło, Iwona Bodys-Cupak, Elżbieta Walewska, Maria Kózka

**Affiliations:** 1 Department of Clinical Nursing, Faculty of Health Sciences, Institute of Nursing and Midwifery, Jagiellonian University Medical College, 31-501 Krakow, Poland; 2 Laboratory of Theory and Fundamentals of Nursing, Faculty of Health Sciences, Institute of Nursing and Midwifery, Jagiellonian University Medical College, 31-126 Krakow, Poland

**Keywords:** BMI, MNA, nutritional status, postoperative complications

## Abstract

In patients scheduled for surgery, nutritional disorders worsen during the perioperative period, which is often a risk factor for postoperative complications. The aim of the study was to determine relationship between the preoperative nutritional status of elderly people with stomach, pancreatic and colon cancer and the incidence of postoperative complications and the length of hospital stay. The study included 143 patients with gastrointestinal cancer, aged 65–68, qualified for surgery. Mini Nutritional Assessment, body mass index questionnaires and medical records were used. Malnutrition was found in 9.8%, and a risk of malnutrition in 53.5% of the respondents. Body mass index showed overweight in 28% and obesity in 14% of the patients. Complications occurred in all types of nutritional status, the most common were those requiring intensive care unit treatment (36.8%), pancreatic and biliary fistulas (29.4%) and surgical site infections (58.2%). Gastric cancer patients at risk of malnutrition stayed longer in the hospital. Postoperative complications and longer hospital stays were observed more frequently in cases of overweight, obesity, malnutrition and its risk. Disturbances in the nutritional status, in the form of malnutrition and its risk, as well as overweight and obesity, determined more frequent occurrence of postoperative complications and longer hospital stay.

## 1. Introduction

In recent years, a large increase in the number of elderly people has been observed. The aging process causes organ dysfunction, which leads, among other things, to the development of multiple diseases. Approximately 75% of elderly people suffer from comorbidities, including cancer, which often require surgical intervention [1]. The aging of the immune system, metabolic disorders, the surgical trauma itself and the patient’s health condition resulting from the cancer process increase the risk of postoperative complications. An additional risk factor for complications may be nutritional disorders in the form of risk or malnutrition, as well as overweight or obesity accompanying metabolic diseases [2]. Overweight or obesity modify the course of many diseases, increasing the risk of complications resulting from anesthesia and surgery [3]. Additionally, malnutrition develops or worsens during hospitalization in surgically treated patients and is a risk factor for postoperative complications, prolongs hospitalization and increases treatment costs [4,5,6,7,8]. Studies show that the greatest probability of malnutrition occurs in patients with cancer diseases (30–90%), inflammatory bowel diseases (80%), in the elderly and in patients with respiratory system diseases and treated neurologically [8]. In people qualified for surgery, especially the elderly due to the high risk of complications, it is important to recognize nutritional disorders early and implement nutritional interventions as soon as possible [8,9,10].

In clinical practice, in the case of patients with gastrointestinal tract cancer, the risk of postoperative complications resulting from nutritional status disorders related to malnutrition is most often taken into account. Scientific publications also rather focus on the results of studies showing malnutrition in gastrointestinal cancer patients. Reports and studies related to the analysis of body mass index (BMI) exceeding the norm in such a group of patients and the potential risks resulting from it are rarely found. Studies on the correlation of obesity and overweight with postoperative complications are particularly lacking. In particular, there are no analyses of patients with cancer disease in which malnutrition and its risk occurs most often. The studied elderly population is also overweight and obese, which may have a similar effect on the patient’s clinical status as malnutrition-related disorders. Hence, the inspiration to conduct and describe this study in this manuscript in order to expand the knowledge in this field. The assessment of the patient’s nutritional status, conducted to identify risk factors for postoperative complications, should be one of the most important elements of preoperative preparation.

It is important to use nutritional assessment tools. In elderly patients, the MNA scale is recommended and commonly used to identify malnourished patients and those at risk of malnutrition [4]. On the other hand, the most frequently used tool for recognizing and confirming overweight and obesity is BMI [11].

The aim of the study was to assess the nutritional status in the preoperative period and to determine the relationship between the nutritional status of elderly people with stomach, pancreatic and colorectal cancer, as well as the incidence of postoperative complications and the length of stay in the hospital.

The hypothesis of the study is that malnutrition, overweight and obesity represent risks of postoperative complications and prolonged hospitalization in elderly patients with gastrointestinal cancer.

## 2. Materials and Methods

The study was conducted among 143 patients (57.3% men and 42.7% women) diagnosed with cancer disease of the gastrointestinal tract at the Clinical Department of General, Oncological, Gastroenterological and Transplant Surgery at the University Hospital in Krakow in the period from August 2018 to July 2019. It included 45 patients with gastric cancer, 48 with pancreatic cancer and 50 with colorectal cancer, aged 65–68 years. The sample size was calculated using G-power 3.0.10 software. The sample size of 143 had a 95% power of detecting a change of 0.25 at an alpha level of 0.05.

The study included people who met the criteria for inclusion in the study group: age ≥ 65 years, diagnosis of gastric, pancreas or colon cancer, elective surgery, consent to participate in the study.

The exclusion criteria were: age under 65, emergency surgery, no confirmation of cancer in histopathological examination, use of neoadjuvant chemotherapy or preoperative radiotherapy. Patients who underwent only exploratory laparotomy due to cancer spreading that prevented resection or bypassing the tumor from being performed were also excluded from the study.

Patients were recruited for the study on the day of admission to hospital for surgery. In total, 156 people were pre-qualified for the study, including 50 patients with gastric cancer, 56 patients with pancreatic cancer and 50 patients with colorectal cancer. All patients had a confirmed diagnosis of cancer by histopathological examination. Each patient underwent a surgical consultation or a multidisciplinary oncology consultation, defining the indications for surgical treatment and the planned scope of surgical treatment. Then, internal and anesthesiological assessment were performed, and in cases of indications, additional specialist consultations were carried out. On the basis of such a standard assessment, patients were qualified for surgical treatment.

The study group consisted of patients with various stages of cancer advancement, which was assessed based on the 5-stage classification: stage 0—cancer in a very early stage of development, pre-invasive CIS (carcinoma in-situ); stage I—cancer in an early stage of development; stage II—moderately advanced cancer; stage III—advanced cancer; stage IV—very advanced cancer with the presence of distant metastases [12]. The final decision as to the type of surgery was made by the surgeon individually for each patient during the operation and based on confirmed histopathological intraoperative examination.

**Figure 1:**
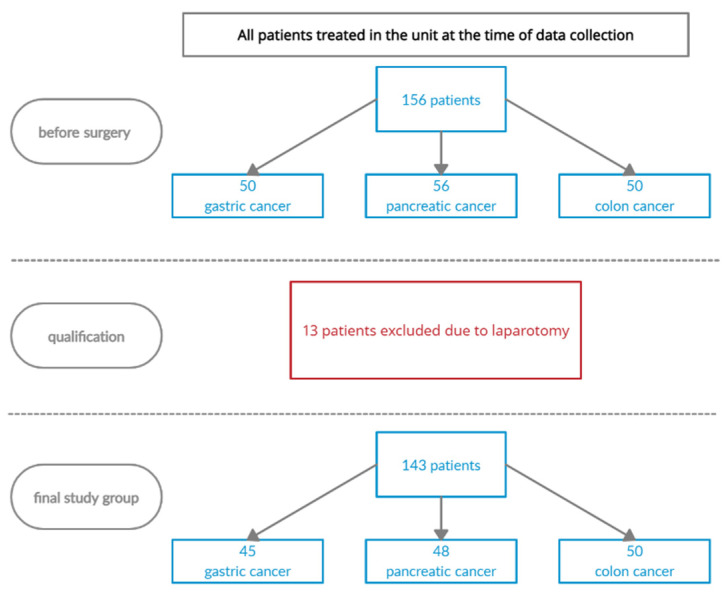
The process of qualifying patients for the study.

After the surgery, based on the exclusion criteria, the study excluded patients whose surgery consisted of an exploratory laparotomy (in the case of gastric cancer—5 patients, pancreatic cancer—8 people). Ultimately, 45 patients with stomach cancer, 48 with pancreatic cancer and 50 with colorectal cancer remained in the study group. The process of qualifying patients for the study is presented in Figure 1.

The analysis of the study results included all patients who were qualified for the study.

The respondents were informed about the purpose of the study, voluntary participation in it and the possibility of withdrawing at each stage, informed consent to participate in the study was obtained. The research procedure was carried out in accordance with the Helsinki Declaration of the World Medical Association [13] and the ethical codes of the Belmont Report [14]. Approval of the Bioethics Committee No. 1072.6120.197.2019 was obtained.

The study was cross-sectional and consisted of describing the variables and analyzing their occurrence and correlation at a given time (pre- and postoperative period): the dependent variable was gastrointestinal cancer (stomach, pancreas and colon) confirmed by histopathology. The independent variables were: nutritional status, occurrence of complications, type of surgery and hospitalization time.

In the preoperative period, the nutritional status was assessed using the Mini Nutritional Assessment (MNA) questionnaire and the BMI was calculated.

The MNA Scale is a multi-dimensional screening tool proven in many clinical conditions. According to the meta-analysis, MNA in the assessment of the nutritional status of patients is characterized by a sensitivity of 96%, specificity of 98% and a predictive value of 97%. The European Society for Clinical Nutrition and Metabolism (ESPEN) recommends its use both as a first-stage screening test and for follow-up test. A screening test was used in the own study. The analysis of nutritional status based on MNA was performed on the basis of the following values: 12–14 points, proper nutritional status; 8–11 points, risk of malnutrition; 0–7 points, malnutrition [15,16,17].

In the analysis of the nutritional status assessment based on BMI, the values according to WHO (World Health Organization) are: <18.5 underweight, 18.5–24.9 normal, 25.0–29.9 overweight, 30.0–34.9 class I obesity, 35.0–39.9 class II obesity, ≥40. 0 III class obesity (morbid) [11].

After the surgery, an analysis of postoperative complications was made from the medical records of the patients.

The study group was divided into two groups depending on the type of surgery (resection or palliative surgery). In the case of gastric cancer, total or partial gastric resection was performed; in the case of pancreatic cancer, Whipple and Traverso pancreatoduedenectomy, peripheral pancreatic resection; and in patients with colorectal cancer, hemicolectomy, abdominoperineal rectal resection and anterior rectal resection were performed. Palliative procedures included gastrointestinal anastomosis, entero-intestinal anastomosis and removal of peritoneal adhesions.

As a result of the analysis of the documentation, the following complications were distinguished: pancreatic and biliary fistulas, surgical site infection (SSI), reoperations (bleeding, anastomotic leak), gastrointestinal motility disorders (obstruction, nausea, vomiting, flatulence), circulatory and respiratory disorders (ABP fluctuations, cardiac arrhythmias, pneumonia, limb edema) and severe cardiopulmonary complications requiring treatment in an intensive care unit (ICU).

The results were analyzed using the STATISTICA 12 PL software. The distribution of qualitative variables was described by the absolute number of individual categories (*n* and their percentage share in the distribution of the variable (%). The relationships between the categorical variables are presented in the form of cross tables. The statistical significance of these relationships was analyzed using the Fisher–Freeman–Halton test. In all the analyses performed, the existence of differences and the strength of the relationship between the variables was estimated at the significance level of *p* < 0.05.

## 3. Results

On the basis of the MNA questionnaire, malnutrition was found in 9.8%, and the risk of malnutrition in 53.5% of the respondents. Malnutrition was more common in the group of people with gastric cancer (17.8%), and the risk of malnutrition in the group of people with pancreatic cancer (62.5%).

The highest percentage of underweight according to BMI was observed in patients with pancreatic cancer, overweight was most often diagnosed in patients with gastric and colorectal cancer and obesity in patients with gastric and pancreatic cancer. Detailed data are presented in Table 1. 

Resection was performed in 109 (76.2%) patients, including 36 (33.0%) with gastric cancer, 28 (25.7%) with pancreatic cancer and 45 (41.3%) with colorectal cancer. Palliative procedures were performed in 34 (23.8%) patients, including 20 (58.8%) procedures in pancreatic cancer patients, 5 (14.7%) in patients with colorectal cancer and 9 in gastric cancer patients (26.5%).

**Figure 2:**
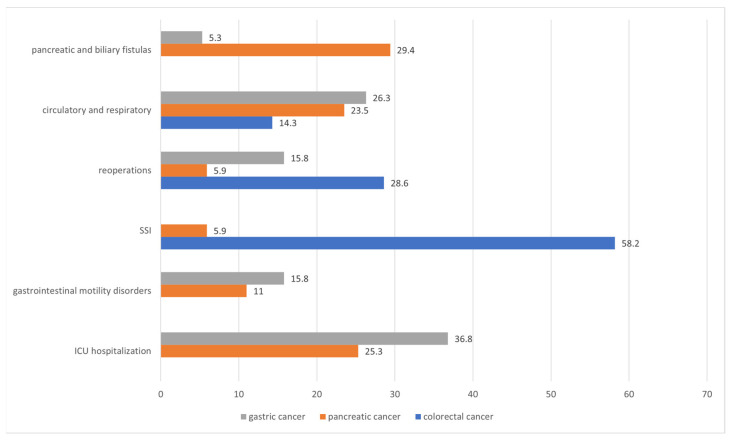
Types of postoperative complications depending on the location of the tumor.

Complications after surgery in the study group varied depending on the type of tumor. In the group with gastric cancer, the most common (36.8%) complications were those that required treatment in the Intensive Care Unit (ICU). In the group with pancreatic cancer, pancreatic and biliary fistulas were the most common (29.4%), and in the group with colorectal cancer, the most common complications were surgical site infections (58.2%). Detailed data are presented in Figure 2.

Statistical analysis showed a relationship between the nutritional status of patients with gastric cancer and the occurrence of postoperative complications, regardless of the type of surgery performed (resection or palliative surgery). Complications occurred more often in the group of patients at risk of malnutrition, those who were malnourished and who were overweight (Table 2).

In the group of patients with pancreatic cancer who underwent palliative procedures, complications were most common among patients at risk of malnutrition. On the other hand, in the case of resection procedures, complications were found more often among overweight and obese patients—Table 3.

When analyzing the results for people with colorectal cancer, it was observed that after palliative surgery, the factors increasing the risk of complications included the risk of malnutrition, overweight and obesity. In the case of resection, complications occurred more often in patients at risk of malnutrition and malnourishment, as well as in overweight and obese patients—Table 4.

In the last stage of the study, an analysis of the relationship between the results of the nutritional status of patients before the surgery in individual study groups and the duration of hospitalization was made. Based on the results of the MNA scale, longer duration of stay was observed in the group of gastric cancer patients at risk of malnutrition. On the other hand, based on the BMI results, in all study groups the hospitalization time was longer in both overweight and obese patients (Table 5).

## 4. Discussion

Proper nutritional status affects the overall functioning of the body, the activity of the immune system and the ability to regenerate. It is particularly important during the convalescence period in people undergoing surgery, mainly due to cancer disease [18]. Malnutrition is the basic issue of patients treated in hospital conditions. The imbalance between energy, nutrient requirements and actual consumption may disrupt the body’s response to environmental stress, including surgery [19].

The group at risk of nutritional disorders, including the risk of malnutrition and malnutrition, are elderly people, in whom such a condition is associated with physiological, metabolic and functional changes, which may lead to many disorders of individual systems and organs [15]. These disorders are associated with increased morbidity, prolonged hospital stay, reduced quality of life and mortality [20,21]. Moreover, the surgery itself may have a negative impact on the nutritional status, especially in geriatric patients [22,23].

Patients undergoing oncological operations on the gastrointestinal tract are at risk of malnutrition as a result of anorexia, restriction in food intake due to diagnosis and treatment, malabsorption or diarrhea [6,9,24,25,26].

A correlation has been demonstrated between disturbed nutritional status and worse perioperative outcomes due to excessive inflammatory response as a result of decreased protein and energy levels after surgical trauma [27].

Studies show that in patients with cancer of the gastrointestinal tract undergoing major surgery, preoperative malnutrition is associated with an increased number of complications. Eating disorders are recognized risk factors for postoperative complications in the adult population [28,29].

Data from the National Surgical Quality Improvement Program (NSQIP) confirm that malnutrition is one of the top ten preoperative risk factors leading to poor outcome in the form of complications or increased mortality [30].

Early identification and treatment of malnutrition is critical to improving patient outcomes. For these reasons, nutritional screening is recommended, even if nutritional risk is not explicitly present at diagnosis. In clinical practice, however, the global consensus regarding the optimal test method remains difficult to define. The issued ESPEN (European Society of Clinical Nutrition and Metabolism) recommendation in 2019 proposed GLIM (Global Leadership Initiative on Malnutrition) criteria that combine phenotypic elements (weight loss, reduced BMI and reduced muscle mass) and etiological elements (reduced food consumption/assimilation and disease burden/inflammation). Based on GLIM, it is recommended that the diagnosis of malnutrition requires a combination of at least one phenotypic criterion and one etiological criterion. In the first stage, however, the malnutrition diagnosis algorithm according to GLIM guidelines indicates the use of the NRS 2002, SGA or MNA scale [31].

The aim of the own research was to determine the relationship between the preoperative nutritional status of elderly people with stomach, pancreatic and colorectal cancer and the incidence of postoperative complications.

In our study, the MNA questionnaire was used for pre-operative assessment of the nutritional status of patients with gastrointestinal cancer as a recommended tool to assess the nutritional status and identify factors influencing the nutritional status of the elderly [14].

In our own research, on the basis of the MNA questionnaire, it was shown that 9.8% of the respondents demonstrated malnutrition, including 17.8% with gastric cancer, 8.3% with pancreatic cancer and 4.0% with colorectal cancer. On the other hand, the risk of malnutrition was found in more than half of the respondents (53.5%), including patients with stomach cancer 48.9%, pancreatic cancer 62.5% and colorectal cancer 50%. Similar results were obtained by Mignini EV et al. among patients who underwent extensive operations on the gastrointestinal tract. Abnormal nutritional status was present in over half (54%) of the respondents (malnutrition in 10% and the risk of malnutrition in 44% of patients, respectively), including the group of people over 80 years of age in which malnutrition was diagnosed in 16.70%, and the risk of malnutrition in 58.3%. Malnutrition significantly correlated with older age (r = 0.03400) [32].

Slightly higher rates of malnutrition were demonstrated by Kim et al. when assessing the nutritional status with the same tool [33]. The authors showed that 25% of patients were malnourished, and the rates of preoperative malnutrition incidence and risk of malnutrition were approximately 87%, respectively. The study by La Torre et al., based on the nutritional screening tools used, showed that over 80% of patients with preoperative cancer diagnosis were classified as moderately and severely malnourished [34].

The results of studies by other authors also confirm the presence of malnutrition in the group of elderly people diagnosed with cancer, identified on the basis of the MNA scale [35,36].

In previous studies carried out in a clinical hospital in Krakow, which assessed the usefulness of elements of the perioperative risk model in people over 65 years of age, abnormal MNA test results were found in 39.2% patients, compared to the group of patients without cancer diagnosis (13.6%), *p* = 0.002. These results showed the potential usefulness of the MNA test in predicting complications related to surgery in a group of oncological patients [37]. This thesis is also confirmed by the similar results of Rizi M. et al. On the occurrence of malnutrition in the group of patients with gastrointestinal cancer [38] and other published studies in which malnutrition was found in patients treated surgically for gastrointestinal cancer [39,40].

The authors of studies conducted in the USA report that perioperative malnutrition remains largely underdiagnosed and untreated, although 24–65% of patients undergoing surgery are at risk of malnutrition or malnourished [41].

Another parameter that was taken into account in our own research when assessing the nutritional status was BMI. The assumption was to try to correlate BMI above and below normal and the complications involved.

In our study, based on BMI analysis, underweight was diagnosed in 6.3% of patients, overweight in 28.0% and obesity in 14.0% of patients. More than 50% of patients showed normal BMI. The highest percentage of underweight based on BMI was observed in patients with pancreatic cancer, overweight was most often diagnosed in patients with gastric and colorectal cancer, and obesity in patients with gastric and pancreatic cancer. In a study by Wightman et al., in 388 patients with esophageal cancer, median age 62 [42], the distribution of BMI was similar in patients with underweight—5.6% and overweight—31.4%, while the results differed in patients with obesity (34.3%) and a normal BMI—28.7%. Another study concerned over 9000 patients with esophageal cancer who had undergone elective surgery [43]. The results varied, underweight was diagnosed in 3% of patients, overweight in 36%, obesity in 29% and 32% of patients had normal BMI values. Over 2500 patients with gastrointestinal diseases participated in a prospective multicenter study, including 22.2% of patients who were obese [44], and in a study in Mexico, among 1430 patients after inguinal hernia surgery, cholecystectomy, appendectomy, 53% of patients were obese or overweight [45].

The above-mentioned studies show how large the variation in the population of patients undergoing surgery is in terms of body mass index.

It is widely believed that being underweight, and malnutrition often associated with it, is a risk factor for postoperative complications. In contrast, obesity increases morbidity and mortality in the population, but there are few studies comparing the incidence of postoperative complications in obese and non-obese subjects [45].

Subsequently, the analysis of the relationship between the nutritional status and the occurrence of complications in the postoperative period was undertaken. The analysis of the results showed that complications occur more often in patients with abnormal body weight—both in the case of the risk of malnutrition and malnutrition as well as overweight and obesity. In our study, the most common complications among people with gastric cancer required treatment in the Intensive Care Unit (36.8%). In patients undergoing pancreatic cancer surgery, the most common complications were pancreatic and biliary fistulas (29.4%), and surgical site infection (58.2%) was the most common complication of colorectal cancer (58.2%). When analyzing the nutritional status on the basis of the MNA scale, in each type of cancer, complications occurred more frequently in the group of patients who were malnourished or at risk of malnutrition compared to patients with normal body weight. Similarly, in the studies of Mignini et al., comparing the complications with the nutritional status determined before the surgery, it was observed that on the third postoperative day, 31% of systemic complications were noted in well-nourished patients, 65.5% in those at risk of malnutrition, and 3.5 % in malnourished patients [32]. Well-nourished patients had complications mostly in the early observation period (3 days postoperatively) but recovered either partially or completely within 6 days postoperatively and malnourished patients had fewer complications on day 3 postoperatively but had a worse outcome 6 days after surgery [32]. A study by Sasaki et al. on the determination of the geriatric nutritional risk index in patients with colorectal cancer showed that its low preoperative value (≤98) was associated with the severity of postoperative complications and poor prognosis in patients aged ≥ 65 years and after surgery. It has been found that the determination of a geriatric nutritional risk index before surgery can be a useful tool to identify a population at high risk of morbidity and mortality among elderly patients [46].

The results of own research are similar to those published in the previous research conducted in Krakow. In the group of patients treated surgically, in the case of malnourished patients, postoperative complications were more frequent (*p* = 0.001) [37], and in another study conducted in a group of patients with gastric cancer, the incidence of complications was observed in 28.3% of patients and the influence of nutritional status on the incidence of complications was also demonstrated; both general and infectious complications were more common in the group of malnourished patients [39]. The results of many other studies also indicate that nutritional disorders, especially malnutrition in cancer patients, are associated with a higher incidence of various complications, including intensive care unit hospitalization, and negative clinical results, which is consequently associated with prolonged hospitalization and increased mortality [47,48,49,50].

Surgical procedures on the abdominal cavity carry a significant risk of postoperative complications, and malnutrition is an additional factor that may affect their occurrence, to which patients are exposed in the perioperative period. Preoperative weight loss adversely affects the survival of patients treated for cancers of the upper gastrointestinal tract or in colorectal surgery [5]. Many studies have confirmed the importance of the period of preparation for surgical procedures in the form of pre-rehabilitation programs, i.e., the use of the ERAS protocol (Enhanced Recovery After Surgery Protocol), an indispensable element of which, among others, is nutritional preparation in perioperative hospital care. It reduces the risk of early complications of surgical treatment, shortens hospitalization time and extends the survival of cancer patients [51,52,53]. Quick and effective monitoring of the nutritional status and implementation of nutritional support in cancer patients maintains or improves the nutritional status, and also improves treatment outcomes [36].

The authors’ own research also analyzed the relationship between the nutritional status of patients before surgery, based on BMI, and the occurrence of postoperative complications as a result of procedures performed in the area of the stomach, pancreas and large intestine. Postoperative complications after resection procedures have been demonstrated in patients whose BMI was normal or indicated that they were overweight or obese before surgery. After gastric surgery, complications occurred in 61.5% of overweight patients and in 43.8% of patients with normal body weight. Similarly, after pancreatic surgery, 66.7% of patients with complications were overweight and 40% were obese. Only after colon surgery, 50% of underweight patients had postoperative complications, as well as 35.7% of overweight patients and 50% of obese patients. The previously mentioned scientists from La Salle University in Mexico City [45] obtained results showing that obese patients had more postoperative complications. At the same time, however, they believe that there is a need to explain this relationship based on a larger group of patients in prospective studies. Studies involving over 2500 patients from 127 centers [44] showed that obese patients are more prone to postoperative complications, but as a result of oncological procedures, while a reduced risk of complications in obese patients was observed after surgical procedures unrelated to diagnosis of cancer.

Wightman et al. obtained different results than in our own research. In the observation of patients after removal of the esophagus, underweight subjects were more prone to postoperative complications and their hospital stay was extended [42]. In overweight and obese patients, the risk of complications was similar to that in patients with normal BMI. A similar group of patients after esophagectomy was observed by Mitzman et al. They found that both patients with low and very high BMI are at risk of postoperative complications [43]. A study from the Netherlands [31] concerned approximately 4300 operated patients and showed that the occurrence of postoperative complications is the main risk factor in surgery in underweight patients, and also that it is unjustified to consider obesity as the main risk factor in general surgery. In the United States of America [35], the influence of increased BMI on the results of spine surgery was investigated in a group of over 7000 patients, 8% of whom were obese. It has been proven that patients diagnosed with obesity did not show an increased rate of complications, reoperation and rehospitalization. In Iran, a study was conducted in a group of 1120 patients undergoing coronary artery bypass surgery. Both patients with low BMI and high BMI were at risk of postoperative complications; however, these complications differed. In the obese group, atelectasis and fever was more frequent, and in the underweight group, breathing difficulties and prolonged reintubation time were observed [36].

In a retrospective study by Eljaz et al. concerning 775 patients undergoing gastrectomy for gastric cancer, BMI alone had no effect on perioperative morbidity, relapse-free survival or overall survival. However, patients with a BMI < 18.5 and low preoperative albumin levels had significantly reduced overall survival after gastrectomy [54].

Our own study also analyzed the length of stay of patients in hospital, depending on their nutritional status. Among patients with gastric cancer who experienced postoperative complications due to malnutrition and the associated treatment in the intensive care unit, hospitalization time was increased, but not reaching statistical significance. This could, however, be due to the small sample size. On the other hand, based on the BMI results, overweight and obese people stayed in the hospital the longest in all study groups. In the Mignini study, older age and nutritional status correlated with longer hospital stay, showing a statistical tendency, which was also explained by the insufficient size of the group participating in the study. Therefore, in this case, there is also a tendency for differences in hospitalization depending on the nutritional status [32]. According to the authors of other studies, it has been shown that the nutritional status associated with malnutrition or the risk of malnutrition has a large impact on postoperative complications and the length of hospital stay, even in minimally invasive conditions [52,55].

Obesity in general surgery is also known to have a negative effect on perioperative complications, longer hospital stays, care costs and even mortality [56,57].

One of the elements of the assessment of the risk of complications and death in the perioperative period, especially in the group of elderly people, is to examine the nutritional status to select a group of patients with abnormalities. Temporarily postponing surgery of these patients so that corrective measures can be taken may allow for safer surgery and postoperative period, especially when the need for cancer surgery is more important than for non-cancerous diseases.

The nutritional status results of surgical patients clearly underline the importance of early diagnosis of the risk of malnutrition and malnutrition itself; focusing on the degraded nutritional status paves the way for a faster and more effective approach to the problem. Therefore, perioperative nutritional support may be important to address one of the major factors affecting both the outcome of surgery and the development of complications and hospitalization time [58,59].

Nutritional therapy in operated patients is aimed at compensating for malnutrition before surgery and maintaining the nutritional status after surgery [60].

In the immediate postoperative phase, nutritional therapy can only minimize muscle catabolism, even providing energy for optimal healing and regeneration. Surgical trauma and possible infection can disrupt the recovery of lean body mass. Severely malnourished patients may show a dynamic form of sepsis, and in such situations nutritional therapy will not help to maintain lean body mass, but may support an adequate stress response, thereby promoting recovery [58,59,60].

The 2017 ESPEN guidelines strongly recommend nutritional intervention prior to major surgery in cases of severe nutritional risk or overt malnutrition, administered for a minimum of 10 to 14 days, even at the cost of delaying surgical intervention [52].

By analyzing the results for the entire study group; factors that increased the risk of complications after surgery were both the risk of malnutrition and malnutrition, as well as overweight and obesity.

The above analysis of preoperative abnormal results of both MNA and BMI tests, showing disturbances in nutritional status and reflecting the occurrence of complications, indicates the likely value and usefulness of determining the nutritional status in the period before surgery in the assessment of perioperative risk in elderly patients with neoplastic diseases. Preoperative malnutrition or obesity may be associated with greater postoperative complications, with prolonged hospital stay, and an even greater risk of re-hospitalization.

The limitation of the study was the observation period during the patients’ stay in the hospital after the surgery. Therefore, further studies are recommended, also on a larger sample of patients, to assess the development of complications even weeks or months after the surgery.

## 5. Conclusions

An observational study of the nutritional status before surgery in patients with gastric, pancreatic and colon cancer revealed nutritional status disturbances in the form of a risk of malnutrition and malnutrition, as well as overweight and obesity. Preoperative malnutrition, as well as overweight and obesity, was associated with more frequent postoperative complications. Complications most often occurred in the group of patients with gastric cancer.

There was also a trend towards longer hospital stays in people at risk of malnutrition with gastric cancer and in other overweight and obese patients.

Due to the observation period limited to patients’ hospital stay after surgery, further studies on a larger sample are recommended to assess the development of complications even weeks or months after surgery.

The test results can be used to undertake preparatory interventions for surgery (prehabilitation) regarding nutritional support determined individually for the patient, as well as taking actions related to the implementation of physical activity.

## Figures and Tables

**Table 1:** Nutritional status of patients before surgery considering the location of the tumor.

Questionnaire/Indicator		*n* = 143			*p*
		Gastric Cancer *n* = 45 (31%)	Pancreatic Cancer *n* = 48 (34%)	Colorectal Cancer *n* = 50 (35%)	
		*n* (%)	*n* (%)	*n* (%)	
**MNA**	Proper nutritional status	15 (33.3)	14 (29.2)	23 (46.0)	0.10
	The risk of malnutrition	22 (48.9)	30 (62.5)	25 (50.0)	
	Malnutrition	8 (17.8)	4 (8.3)	2 (4.0)	
**BMI**	Underweight	2 (4.4)	5 (10.4)	2 (4.0)	0.52
	Standard	21 (46.7)	26 (54.2)	27 (54.0)	
	Overweight	15 (33.3)	9 (18.8)	16 (32.0)	
	I class obesity	7 (15.6)	7 (14.6)	5 (10.0)	
	II class obesity	0 (0.0)	1 (2.1)	0 (0.0)	

*n*—number of respondents, *p*—significance level for the Fischer–Freeman–Halton test, BMI—body mass index, MNA—Mini Nutritional Assessment.

**Table 2:** The nutritional status of patients with gastric cancer before surgery and the incidence of postoperative complications considering the type of surgery.

Gastric Cancer Patients *n* = 45										
	Palliative Surgery					Resection Surgery				
Questionnaire/ Indicator	No complications *n* = 6 (13.3%)		Occurrence of Complications *n* = 3 (6.7%)		*p*	No Complications *n* = 20 (44.4%)		Occurrence of Complications *n* = 16 (35.6%)		*p*
**MNA**	***n***	**%**	***n***	**%**		***n***	**%**	***n***	**%**	
Normal nutritional status	1	100.0	0	0	0.57	9	64.3	5	35.7	0.38
Risk of malnutrition	2	50.0	2	50.0		10	55.7	8	44.0	
Malnutrition	3	75.0	1	25.0		1	25.0	3	75.0	
**BMI**										
Underweight	2	100	0	0	0.14	0	0	0	0	0.25
Standard	4	80.0	1	20.0		9	56.3	7	43.8	
Overweight	0	0	2	100.0		5	38.5	8	61.5	
I class obesity	0	0	0	0		6	85.7	1	14.3	
II class obesity	0	0	0	0		0	0	0	0	

*n*—number of respondents, *p*—significance level for the Fischer–Freeman–Halton test, BMI—body mass index, MNA—Mini Nutritional Assessment.

**Table 3:** Nutritional status of patients with pancreatic cancer before surgery and the incidence of postoperative complications considering the type of surgery.

Pancreatic Cancer Patients *n* = 48										
	Palliative Surgery					Resection Surgery				
Questionnaire/ Indicator	No Complications *n* = 14 (29.2%)		Occurrence of Complications *n* = 6 (12.5%)		*p*	No Complications *n* = 17 (35.4%)		Occurrence of Complications *n* = 11 (22.9%)		*p*
**MNA**	***n***	**%**	***n***	**%**		***n***	**%**	***n***	**%**	
Normal nutritional status	3	100.0	0	0	0.24	7	63.6	4	36.4	0.93
Risk of malnutrition	9	60.0	6	40.0		9	60.0	6	40.0	
Malnutrition	2	100.0	0	0		1	50.0	1	50.0	
**BMI**										
Underweight	2	66.7	1	33.3	0.82	2	100.0	0	0	0.49
Standard	7	63.6	4	36.4		10	66.7	5	33.3	
Overweight	2	66.7	1	33.3		2	33.3	4	66.7	
I class obesity	2	100.0	0	0		3	60.0	2	40.0	
II class obesity	1	100.0	0	0		0	0	0	0	

*n*—number of respondents, *p*—significance level for the Fischer–Freeman–Halton test, BMI—body mass index, MNA—Mini Nutritional Assessment.

**Table 4:** Nutritional status of patients with colorectal cancer before surgery and the incidence of postoperative complications considering the type of surgery.

Colorectal Cancer Patients *n* = 50										
	Palliative Surgery					Resection Surgery				
	No Complications *n* = 3 (6.0%)		Occurrence of Complications *n* = 2 (4.0%)		*p*	No Complications *n* = 33 (66.0%)		Occurrence of Complications *n* = 12 (24.0%)		*p*
**MNA**	***n***	**%**	***n***	**%**		***n***	**%**	***n***	**%**	
Normal nutritional status	1	50.0	1	50.0	0.93	14	66.7	7	33.3	0.40
Risk of malnutrition	2	66.7	1	33.3		18	81.8	4	18.2	
Malnutrition	0	0	0	0		1	50.0	1	50.0	
**BMI**										
Underweight	0	0	0	0	0.40	1	50.0	1	50.0	0.29
Standard	2	100.0	0	0		21	84.0	4	16.0	
Overweight	1	50.0	1	50.0		9	64.3	5	35.7	
I class obesity	0	0	1	100.0		2	50.0	2	50.0	
II class obesity	0	0	0	0		0	0	0	0	

*n*—number of respondents, *p*—significance level for the Fischer–Freeman–Halton test, BMI—body mass index, MNA—Mini Nutritional Assessment.

**Table 5:** Relationship between the nutritional status of the respondents before surgery and the length of hospitalization, taking into account the type of cancer.

	Gastric Cancer			Colorectal Cancer			Pancreatic Cancer		
	M	SD	*p*	M	SD	p	M	SD	*p*
**MNA**									
Proper nutritional status	9.7	8.2	0.15	8.8	4.6	0.39	10.8	7.3	0.18
Risk of malnutrition	14.3	16.2		8.7	5.0		7.6	4.8	
Malnutrition	9.3	7.5		8.5	4.7		5.5	0.7	
**BMI**									
Underweight	6.5	2.1	0.74	8.8	4.4	0.86	5.5	0.7	0.16
Standard	9.3	5.8		8.6	5.2		7.7	4.8	
Overweight	14.7	17.0		9.2	5.5		9.6	5.3	
I class obesity	15.4	18.3		9.3	3.4		15.4	11.8	

M—mean, SD—standard deviation, *p*—significance level for the Fischer-Freeman-Halton test, BMI—body mass index, MNA—Mini Nutritional Assessment.

## Data Availability

The datasets generated during and/or analyzed during the current study are available from the corresponding author upon reasonable request.
